# Genome-wide analysis of DNA methylation in Hirschsprung enteric precursor cells: unraveling the epigenetic landscape of enteric nervous system development

**DOI:** 10.1186/s13148-021-01040-6

**Published:** 2021-03-09

**Authors:** Leticia Villalba-Benito, Daniel López-López, Ana Torroglosa, Carlos S. Casimiro-Soriguer, Berta Luzón-Toro, Raquel María Fernández, María José Moya-Jiménez, Guillermo Antiñolo, Joaquín Dopazo, Salud Borrego

**Affiliations:** 1 Department of Maternofetal Medicine, Genetics and Reproduction, Institute of Biomedicine of Seville (IBIS), University Hospital Virgen del Rocío/CSIC/University of Seville, 41013 Seville, Spain; 2 Centre for Biomedical Network Research on Rare Diseases (CIBERER), 41013 Seville, Spain; 3 Clinical Bioinformatics Area, Fundación Progreso y Salud (FPS), CDCA, University Hospital Virgen del Rocío, 41013 Sevilla, Spain; 4 Computational Systems Medicine, IBIS, University Hospital Virgen del Rocío/CSIC/University of Seville, 41013 Seville, Spain; 5 Department of Pediatric Surgery, University Hospital Virgen del Rocío, 41013 Seville, Spain

**Keywords:** Hirschsprung disease, Whole genome bisulfite sequencing, DNA methylation, Enteric nervous system development, Epigenetic regulation

## Abstract

**Background:**

Hirschsprung disease (HSCR, OMIM 142623) is a rare congenital disorder that results from a failure to fully colonize the gut by enteric precursor cells (EPCs) derived from the neural crest. Such incomplete gut colonization is due to alterations in EPCs proliferation, survival, migration and/or differentiation during enteric nervous system (ENS) development. This complex process is regulated by a network of signaling pathways that is orchestrated by genetic and epigenetic factors, and therefore alterations at these levels can lead to the onset of neurocristopathies such as HSCR. The goal of this study is to broaden our knowledge of the role of epigenetic mechanisms in the disease context, specifically in DNA methylation. Therefore, with this aim, a Whole-Genome Bisulfite Sequencing assay has been performed using EPCs from HSCR patients and human controls.

**Results:**

This is the first study to present a whole genome DNA methylation profile in HSCR and reveal a decrease of global DNA methylation in CpG context in HSCR patients compared with controls, which correlates with a greater hypomethylation of the differentially methylated regions (DMRs) identified. These results agree with the de novo Methyltransferase 3b downregulation in EPCs from HSCR patients compared to controls, and with the decrease in the global DNA methylation level previously described by our group. Through the comparative analysis of DMRs between HSCR patients and controls, a set of new genes has been identified as potential susceptibility genes for HSCR at an epigenetic level. Moreover, previous differentially methylated genes related to HSCR have been found, which validates our approach.

**Conclusions:**

This study highlights the relevance of an adequate methylation pattern for a proper ENS development. This is a research area that provides a novel approach to deepen our understanding of the etiopathogenesis of HSCR.

**Graphic abstract:**

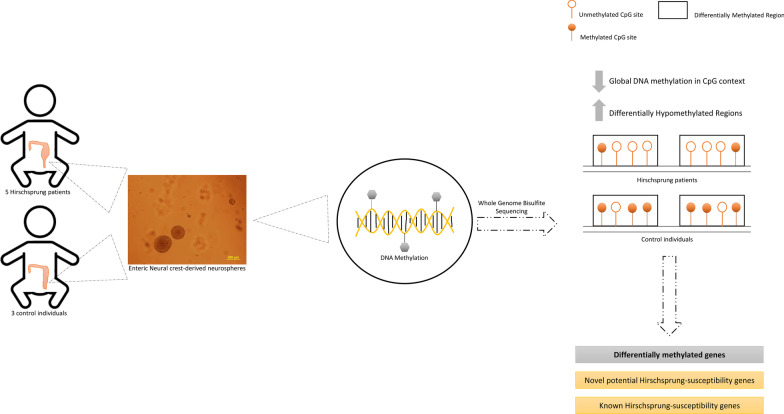

**Supplementary Information:**

The online version contains supplementary material available at 10.1186/s13148-021-01040-6.

## Background

Hirschsprung disease (HSCR, OMIM 142,623) or aganglionic megacolon, is a neurocristopathy affecting 1:5000 newborns. HSCR is characterized by a variable length aganglionosis along a variable length of the distal bowel, resulting in severe intestinal dysfunction [1]. The disease appears either on a familial basis or sporadically showing a complex inheritance pattern with low, sex dependent penetrance and variable expression, and may be associated with other developmental defects [2]. Based on the length of the aganglionic region, HSCR phenotypes are classified as: short-segment forms (S-HSCR), which include patients with aganglionosis as far as the splenic flexure, long-segment forms (L-HSCR), in which aganglionosis extends beyond the splenic flexure and total colonic aganglionosis forms (TCA) [3]. Such aganglionosis is caused by failures in the proliferation, migration, differentiation and/or survival of the enteric precursor cells (EPCs) derived from neural crest cells (NCCs), which avoid an optimal colonization of the gastrointestinal tract during embryonic Enteric Nervous System (ENS) development.

EPCs can be isolated from human postnatal intestinal tissue and constitute a robust tool to study the mechanisms implicated in the ENS and HSCR. These cells grow in clusters known as neurosphere-like bodies (NLBs) and include stem cells with their progeny derived from the neural crest. It has been described that EPCs contained in the NLBs can be transplanted into the aganglionic intestine to restore their contractile properties [4, 5]. In addition, in previous studies we validated EPCs as a useful tool for the study of the ENS and HSCR through different methodological approaches [6, 7].

ENS formation requires a series of complex cellular events that are guided by specific molecular signals involving both EPCs and intestinal environment, which are finally regulated by a particular gene expression pattern [8]. Accurate coordination of these processes is critical, and failures throughout them may lead to HSCR. For this reason, this pathology is considered as a polygenic or complex disease. Extensive research over the past few decades has provided important insights about the causes that contribute to the onset of HSCR, including both genetic and epigenetic factors [2, 9]. Regarding genetic factors, a wide spectrum of genetic variants that affect diverse genes has been associated with HSCR, being the *RET proto-oncogene* the major disease-causing gene. Nevertheless, the genetic cause of the disease in a large portion of HSCR patients remains unknown [2]. Therefore, a more comprehensive global perspective of the molecular basis of HSCR is demanded. In this respect, the high genetic heterogeneity of HSCR patients might be unraveled by the identification of new processes involved in HSCR onset, such as epigenetic mechanisms. Thanks to the development and implementation of high throughput sequencing methods, this kind of mechanisms can be determined like never before.

Epigenetic mechanisms are acquiring increasing evidence of playing a major role in HSCR [9, 10]. In this sense, DNA methylation by the DNA methyltransferases (DNMT1, DNMT3a and DNMT3b) is a well-known, heritable, and reversible epigenetic modification essential for several cellular processes. DNMT1 represents the maintenance methyltransferase and DNMT3a and DNMT3b act as de novo methyltransferases establishing the methylation pattern during embryogenesis. It has been shown that both de novo methyltransferases are essential for a proper NCCs development [11–13], and that DNMT3b may be regulating ENS development through DNA methylation in the neural crest cells, suggesting that aberrant methylation patterns may play a relevant role in HSCR. Specifically, a lower expression level of *DNMT3b* was detected in EPCs from HSCR patients, which led to a decrease of the global DNA methylation and the overexpression of DNMT3b target genes in these cells, as previously was described by our group [14, 15]. Moreover, a role of DNMT3b in the regulation of the cell cycle by P21-P53 activity in EPCs was reported [7]. In addition, aberrant DNA methylation patterns affecting HSCR susceptibility genes have already been described (*RET, GFRA4, EDNRB, PHOX2B)* [16–19]. Therefore, comprehensive genome-wide DNA methylation maps may shed light on the role of this epigenetic mechanism in the normal ENS development and in HSCR. With this goal, in the present study we have determined the methylation pattern required for the proper ENS development through a Whole-Genome Bisulfite Sequencing assay on EPCs from HSCR patients and control individuals. We have performed a differentially methylated regions (DMRs) analysis, which has allowed us to identify a set of genes potentially controlled by dynamic changes of DNA methylation on clusters of CpGs during ENS development, as well as potentially implicated in HSCR onset through this epigenetic mechanism.

## Results

### HSCR-patients exhibit significantly different methylation patterns in a CpG context

**Fig. 1:**
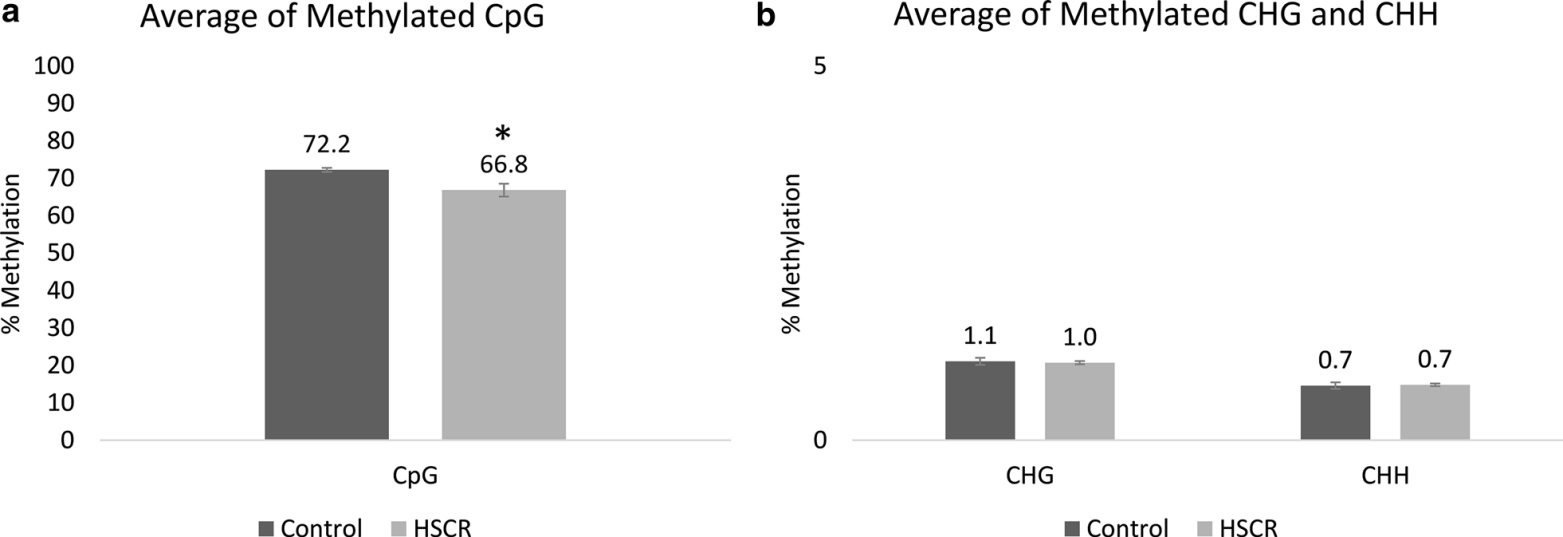
Methylated cytosine percentage in HSCR and control EPCS. **a** Methylated cytosine percentage at CpG context. **b** Methylated cytosine percentage at CHG and CHH contexts. *p < 0·05

To investigate genome-wide DNA methylation patterns at single base pair resolution in HSCR patients, we generated WGBS data from five HSCR cases and three controls. These HSCR patients were selected because the genetic cause of the disease has not been detected or because it was necessary to study other factors involved in the disease to explain the HSCR phenotype. A total of 294 × 10^6^ reads was obtained, of which 80.48% (236 × 10^6^) were aligned to the reference genome. Alignment information is reported in Additional file 1: Table S1. As a result, an average of 788 × 10^6^ cytosines susceptible to methylation was analyzed per sample, which can be found in different nucleotide contexts (CpG, CHG or CHH). In this sense, 4.66% of all cytosines susceptible to methylation were methylated. In agreement with our results, where we found a higher frequency of methylation in CpG context, DNA methylation occurs more frequently in CpG context than in non-CpG context. In HSCR patients, cytosines in CpG context displayed a lower degree of methylation compared to controls (*p* value 0.0318). Significant differences in mean methylation were detected in HSCR patients compared to control. HSCR patients had an average of 66.8% of all cytosines within CpG context methylated, whereas controls had 72.2% of their cytosines methylated (Fig. 1a). In contrast, no changes in DNA methylation were observed in CHG and CHH context (Fig. 1b). A methylation summary of all samples is shown in Table 1.

**Table 1:** Number of methylated and unmethylated cytosines in different cytosine contexts in all individuals analyzed

	C´s in CpG			C´s in CHG			C´s in CHH		
	Methylated	Unmethylated	Methyl-Percentage	Methylated	Unmethylated	Methyl-Percentage	Methylated	Unmethylated	Methyl-Percentage
Control 1	30,050,265	12,167,840	71.18	1,875,285	179,873,431	1.03	4,413,390	551,578,346	0.79
Control 2	42,090,795	15,969,095	72.5	2,427,147	243,349,625	0.99	4,814,247	736,235,146	0.65
Control 3	29,493,831	10,880,970	73.05	1,942,648	167,719,335	1.15	3,745,346	498,358,157	0.75
HSCR 1	26,349,464	13,484,402	66.15	1,747,722	165,419,793	1.05	3,449,030	494,957,852	0.69
HSCR 2	25,597,701	11,573,819	68.86	1,571,162	158,245,790	0.98	3,528,819	489,494,356	0.72
HSCR 3	34,443,644	16,189,793	68.03	2,056,259	205,334,287	0.99	4,515,296	613,140,567	0.73
HSCR 4	31,488,581	13,139,219	70.56	1,946,809	178,421,845	1.08	4,014,891	523,242,181	0.76
HSCR 5	26,623,246	17,308,814	60.6	1,922,228	175,652,489	1.08	4,181,687	519,142,086	0.8

### Identification of differentially methylated regions reveals HSCR-specific methylation patterns

**Fig. 2:**
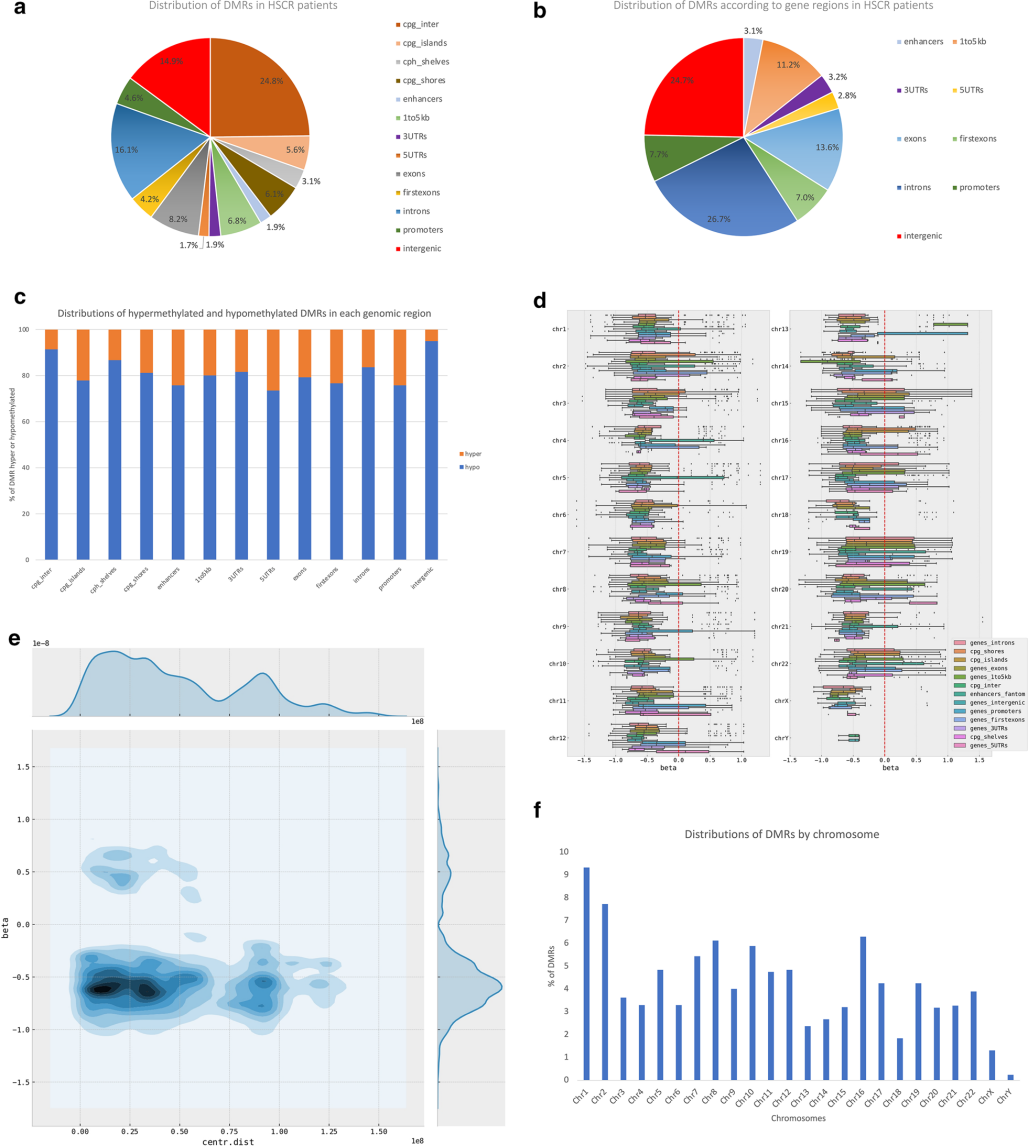
The whole genome DNA methylation landscape of HSCR patients. **a**, **b** Distribution of DMRs throughout each genomic region and across gene elements, respectively. **c** Distribution of hypermethylated and hypomethylated DMRs in each genomic region. **d** DNA methylation level by annotation type throughout each chromosome. The red dashed line represents an unaltered methylation state (ß = 0). Negative values of ß represent hypomethylated DMRs, whereas positive values of ß represent hypermethylated DMRs. **e** DNA methylation level by the distance of the centromere. **f** Distribution of DMRs according to chromosomes

DMRs, which comprise clusters of methylated CpG sites, have considerable implications for disease compared with single CpG sites [20]. Therefore, to gain further insight into the biological relevance of DNA methylation in HSCR context, DMRs in patients were identified, as well as their relation to different genomic regions. As a result, a total of 3363 DMRs were identified, including 2953 DMRs hypomethylated and 410 DMRs hypermethylated (Additional file 2: Table S2). The distribution of DMRs overlapped mainly with CpG_inter (24.8%), introns (16.1%) and intergenic (14.9%), being the DMRs overlapped with introns the most frequent localization according to gene regions (Fig. 2a, b). Using ß value as a proxy of DNA methylation level, we identified a marked presence of hypomethylated DMRs compared to hypermethylated regions in all genomic regions as well as in all chromosomes (Fig. 2c–d). In addition, we found that most DMRs were located close to the centromere region (Fig. 2e). We also examined the distribution of DMRs by chromosomes, and we observed that DMRs are distributed across all chromosomes. Chromosomes 1 and 2 had the highest presence of DMRs (Fig. 2f). Due to the random X-chromosome inactivation events in female cells, we assume that there might be more background noise in the analysis of the sexual chromosomes, which would allow fewer DMRs to be detected. Therefore, in general, these DMRs are hypomethylated, which agrees with the lower DNA methylation level detected at single cytosine resolution.

### GO term enrichment in DMRs based on different gene region locations

Since DNA methylation in different genomic regions exerts different influences on gene activities, we carried out a Gene Ontology enrichment analysis by Metascape to explore DMRs located in different genomic regions. Interestingly, embryonic morphogenesis (GO:0048598) was a GO Biological process shared among the DMRs located within 1 to 5 kb, promoters, exons, and introns. Specifically, the DMRs overlapping with 1 to 5 kb are related to GO terms associated with embryonic and anatomical morphogenesis (GO:0048729, GO:0060485, GO: 2000027) and regulation of neuron differentiation and apoptosis (GO:0045664, GO:0045665, GO:0043523). Promoters are related to regulation of apoptotic processes (GO:0043524, GO:0040365), exons to cell signaling (GO:0007169, GO:0007264, GO:0018108, GO:0098662, GO:0043087), and intra and extracellular organization (GO:0051129, GO:0043062, GO:0030036), and lastly, introns to synapsis and dendritic associated processes (GO:0050808, GO:0050804, GO:0016358), and cell signaling (GO:0007264, GO:0043087 GO:0098662, GO:007169, GO:1905114, GO:0034762) (Additional file 3: Table S3). These results show that the distribution of DMRs is not random, but there is an enrichment across biologically relevant genomic regions.

### Comprehensive analysis of the differentially methylated genes in HSCR patients

**Fig. 3:**
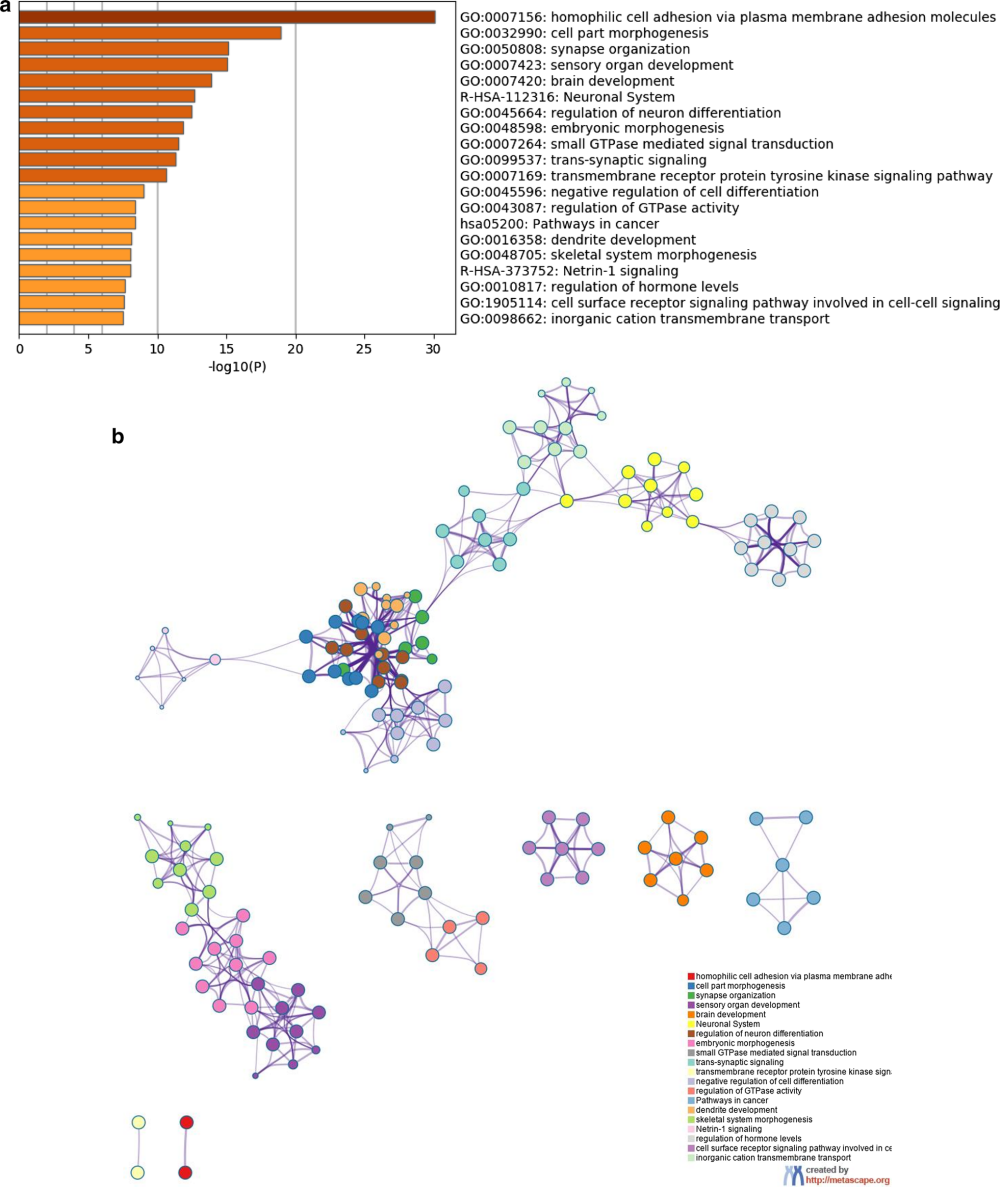
Statistically significant enriched biological processes of DMRs genes in HSCR patients. **a** Histogram that shows the Gene enrichment analysis of genes with DMRs in HSCR patients *versus* controls. **b** Enrichment network where each term is represented by a circle node, where its size is proportional to the number of input genes fall into that term, and its color represents its cluster identity (i.e., nodes of the same color belong to the same cluster). Terms with a similarity score > 0.3 are linked by an edge (the thickness of the edge represents the similarity score). The network is visualized with Cytoscape (v3.1.2) with “force-directed” layout and with edge bundled for clarity. Gene list included into the main and largest network as well as those genes included into the embryonic morphogenesis biological process were included into a list of promising candidates according to their biological processes

We studied methylation based on annotation to genes to gain further insight into the methylation state in susceptibility genes for HSCR already known, as well as to identify new potential susceptibility factors for HSCR. As a result, 1683 genes were identified, including 1345 hypomethylated genes and 338 hypermethylated genes (Additional file 4: Table S4). There are also some DMRs overlapping miRNA and lncRNA (45 and 151, respectively), indicating that DNA methylation may affect ENS development by regulating other epigenetic factors. Next, we performed a functional enrichment analysis using Metascape to assess whether the DMRs related genes were functionally linked to enteric ganglia loss. Interestingly, we found GO terms and pathways implicated in Nervous System developmental processes, such as homophilic cell adhesion (GO:007156), cell part morphogenesis (GO:0032990), synapse organization (GO:0050808), brain development (GO:007420), Neuronal System (R-HSA-112316), regulation of neuron differentiation (GO:0045664), embryonic morphogenesis (GO:0048598), trans-synaptic signaling (GO:0099537), negative regulation of cell differentiation (GO:0045596), dendrite development (GO:0016358), among others enriched terms (Fig. 3a). This result supports that aberrant DNA methylation in genes that controls Nervous System developmental processes such as neurogenesis, synapsis and cell adhesion may contribute to HSCR onset. This analysis allowed us to obtain the network of enriched ontology clusters, where two main networks were detected (Fig. 3b). The main network is related to neural development, including regulation of neuron differentiation (GO:0045664), dendrite development (GO:0016358), cell part morphogenesis (GO:0032990), synapse organization (GO:0050808), trans-synaptic signaling (GO:0099537), regulation of hormone level (GO:0010817), inorganic cation transmembrane transport (GO:0098662), negative regulation of cell differentiation (GO:0045596) and Neural System (R-HSA-112316). The other main network is mainly related to embryonic morphogenesis (GO:0048598). A set of genes were grouped into these categories, which are key functions for ENS development. Therefore, these genes may be considered as promising candidates according to their biological processes (Additional file 5: Table S5).

We then wondered if some of the genes with DMRs had an implication already known in ENS or HSCR onset. With this aim, an automatic bibliographic search of their involvement in such processes for these genes, as well as non-automatic review of the outcomes was performed. Consequently, 55 of these genes have been found to be associated with the manifestation of HSCR and 31 to ENS. It should be noted that 41 and 24 of the genes previously related to HSCR onset and ENS, respectively, were included in the group of genes considered as promising candidates based on their GO analysis, which strengthens the candidacy of these genes as potential genes associated with the pathology (Additional file 5: Table S5). In order to clarify the methylation pattern required for correct ENS development, we analyzed in detail the DNA methylation level within these 55 susceptibility genes for HSCR already known. Consequently, 40 of these genes were hypomethylated and the remaining 15 were hypermethylated in HSCR patients. It should be mentioned the identification of well-known susceptibility genes for HSCR as hypomethylated genes, such as *GDNF*, *GFRA1* and *SOX8*, or hypermethylated such as *ECE1* (Table 2).

**Table 2:** HSCR-susceptibility genes that showed an aberrant methylation level in HSCR patients *versus* controls

	Gene	Seqnames	Start	End	Width	Beta	Pval	Annot,strand	1to5kb	5UTRs	Promoters	Firstexons	Exons	Introns	3UTRs
1	***ABO***	chr9	133,259,941	133,260,919	979	− 0.858	0.004	−						X	
2	*ACTN4*	chr19	38,647,739	38,647,796	58	0.119	0.033	+		X	X	X	X		
3	*ADORA2A*	chr22	24,427,450	24,427,589	140	0.378	0.036	+	X	X	X	X	X	X	
4	***AES***	chr19	3,057,417	3,059,794	2378	− 0.885	0.001	−	X	X	X	X	X	X	
5	***AFAP1-AS1***	chr4	7,768,658	7,771,115	2458	− 0.647	0.047	+						X	
6	***AHNAK***	chr11	62,538,664	62,539,087	424	− 0.987	0.042	−	X					X	
7	***AP3B2***	chr15	82,703,145	82,703,810	666	− 0.526	0.027	−	X					X	
8	***ARNT2***	chr15	80,435,195	80,437,732	2538	− 0.544	0.047	+	X					X	
9	*ASCL1*	chr12	102,958,457	102,958,690	234	0.124	0.023	+				X	X		
10	*BCL11B*	chr14	99,271,192	99,272,182	991	0.123	0.031	−		X	X	X	X		
11	***CA3***	chr8	85,423,540	85,424,639	1100	− 0.669	0.040	+						X	
12	***CACNA1C***	chr12	2,230,284	2,230,376	93	− 1.031	0.003	+						X	
13	*CAPN10*	chr2	240,589,510	240,589,844	335	0.692	0.039	+	X		X		X	X	
14	***CHRNA7***	chr15	32,060,846	32,060,974	129	− 0.645	0.022	+						X	
15	***COL6A1***	chr21	45,992,053	45,994,972	2920	− 0.258	0.046	+	X				X	X	
16	***CREBBP***	chr16	3,773,849	3,774,877	1029	− 0.530	0.043	−	X		X	X	X	X	
17	*CTNS*	chr17	3,635,527	3,637,759	2233	0.345	0.014	+	X	X	X	X	X	X	
18	***DBH***	chr9	133,642,000	133,642,886	887	− 0.636	0.039	+					X	X	
19	*DHCR7*	chr11	71,440,414	71,442,189	1776	0.413	0.026	−	X		X	X	X	X	
20	***DSCAM***	chr21	40,197,142	40,198,150	1009	− 0.805	0.001	−						X	
21	*ECE1*	chr1	21,290,149	21,290,245	97	0.090	0.039	−		X		X	X	X	
22	***FAT3***	chr11	92,451,580	92,452,675	1096	− 0.605	0.008	+						X	
23	***FGD2***	chr6	37,025,970	37,026,290	321	− 0.411	0.050	+						X	
24	***GDNF***	chr5	37,834,936	37,835,683	748	− 0.649	0.012	−		X	X	X	X	X	
25	***GFRA1***	chr10	116,112,901	116,113,661	761	− 0.479	0.043	−						X	
26	*GLI2*	chr2	120,987,959	120,988,503	545	0.176	0.039	+					X	X	X
27	***IHH***	chr2	219,055,465	219,055,534	70	− 0.558	0.038	−					X		
28	*IRS1*	chr2	226,796,575	226,797,233	659	0.983	0.019	−	X			X	X		
29	***ITGB2***	chr21	44,886,345	44,888,733	2389	− 0.637	0.006	−				X	X	X	X
30	***ITIH5***	chr10	7,602,000	7,602,962	963	− 0.737	0.023	−						X	
31	***KCNH2***	chr7	150,958,459	150,958,537	79	− 0.615	0.019	−	X				X	X	
32	***KCNN3***	chr1	154,790,536	154,791,444	909	− 0.735	0.025	−						X	
33	***LAMA1***	chr18	7,109,731	7,109,954	224	− 0.413	0.044	−						X	
34	***MEG3***	chr14	100,824,290	100,824,355	66	− 1.330	0.001	+	X					X	
35	***MUC4***	chr3	195,778,818	195,778,991	174	− 0.499	0.016	−					X	X	
36	***NLRP12***	chr19	53,809,751	53,810,198	448	− 0.729	0.025	−	X				X		
37	***NLRP3***	chr1	247,430,893	247,431,526	634	− 1.075	0.001	+						X	
38	***NOTCH1***	chr9	136,507,039	136,507,083	45	− 0.598	0.038	−	X					X	
39	***NR2F1***	chr5	93,588,640	93,588,660	21	− 1.076	0.004	+	X					X	
40	***NTF3***	chr12	5,452,375	5,453,552	1178	− 0.547	0.017	+	X					X	
41	***NTRK3***	chr15	88,059,303	88,061,553	2251	− 0.788	0.031	−						X	
42	***PCDHA9***	chr5	140,877,089	140,877,155	67	− 0.548	0.016	+						X	
43	*PTCH1*	chr9	95,504,586	95,505,498	913	0.835	0.041	−	X					X	
44	*RARB*	chr3	25,380,243	25,380,308	66	0.959	0.007	+						X	
45	***RBP3***	chr10	47,348,275	47,349,153	879	− 0.821	0.006	+		X	X	X	X		
46	***RBPMS***	chr8	30,441,141	30,441,979	839	− 0.696	0.039	+	X		X			X	
47	***RPS6KA3***	chrX	20,265,270	20,265,640	371	− 0.488	0.007	−						X	
48	*SH2B1*	chr16	28,862,077	28,862,273	197	0.647	0.046	+	X		X			X	
49	***SIX2***	chr2	45,012,355	45,013,494	1140	− 0.739	0.006	−	X			X	X		
50	*SLC2A1*	chr1	42,940,264	42,941,301	1038	0.528	0.043	−						X	
51	***SOX8***	chr16	978,998	980,273	1276	− 0.789	0.008	+	X						
52	***TERT***	chr5	1,292,813	1,293,293	481	− 0.639	0.032	−						X	
53	***TNC***	chr9	115,085,688	115,086,564	877	− 0.600	0.014	−					X	X	
54	***TST***	chr22	37,024,548	37,024,766	219	− 0.305	0.038	−	X						
55	*ZFHX3*	chr16	72,947,027	72,948,255	1229	0.937	0.041	−						X	

Genes in bold were hypomethylated (ß < 0), whereas the remaining genes were hypermethylated (ß > 0)

## Discussion

Studies dedicated to provide a detailed map of epigenomes in normal and pathogenic states are crucial to understand many human diseases. This study supplies valuable insights into HSCR pathogenesis, including the first comprehensive DNA methylation analysis of HSCR patients. Consequently, a lower DNA methylation located in CpG context was detected in HSCR patients EPCs, which correlates with the downregulation of *DNMT3b* and lower global methylation level detected by a colorimetric method previously described in HSCR patients [14]. In addition, according to the literature, most DNA methylation was detected on CpG sites, although methylation in non-CpG context was also detected. DNA methylation in non-CpG context has been described in embryonic stem cells and plays a role in the maintenance of the pluripotent state [21]. Therefore, it is expected that such methylation may also occurs in EPCs.

Only CpG sequences have been used to detect DMRs. We identified 3363 DMRs from HSCR patients, and most of them were hypomethylated. These DMRs overlapped with 1683 genes, and interestingly, these genes were significantly enriched in functions and pathways related to several neural developmental processes. This suggests that DNA methylation influences the regulation of genes with a role in essential processes for proper ENS development. Among the genes with DMRs, 55 and 31 genes have previously been associated with HSCR onset and ENS, respectively. Most of them were included in the gene list of promising candidates according to the enrichment analysis, suggesting the efficiency of the approach carried out to identify potential HSCR-associated genes. Together, these data showed novel HSCR-related changes in DNA methylation that further support an important role for epigenetics in ENS development. Hence, it can be postulated that aberrant methylation patterns in genes with a major role during this process may contribute to a dysfunction of these genes, and ultimately to HSCR onset.

It should be mentioned that most DMRs were located close to the centromeric regions. Centromeres are built from repetitive sequences and transposable elements, and DNA methylation at these regions is essential for the establishment and maintenance of genomic stability [22]. Loss of centromeric methylation may cause an increased rate of recombination of centromeric repeats, displacement of methyl-binding proteins, an increased production of repetitive sequences leading to genomic instability, and chromosome segregation errors [22]. An example of the importance of centromeric DNA methylation in maintaining genomic stability is ICF (Immunodeficiency, Centromeric instability, Facial anomalies syndrome). ICF is a rare autosomal recessive disease characterized by a lack of DNMT3b activity, which causes a DNA methylation depletion, which further leads to reactivation of transposons [22]. The reduced DNMT3b mRNA and protein levels detected in HSCR-EPCs [14], in addition to DNA hypomethylation detected around centromeric regions in the current study, suggest that alterations at this level could be contributing to HSCR onset.

DNA methylation is frequently described as a silencing label, however, several studies have shown that its function changes with genomic context and is more complex than we thought at first [23, 24], even may depend on transcript type, expression level and distance from the transcription start site [25]. Therefore, understanding the functions of DNA methylation is necessary for interpreting changes in DNA methylation in diseases.

DMRs overlapping with genes bodies (gene region past the first exon) may have important roles in transcription elongation and alternative splicing [24]. Moreover, several studies suggest that DNA methylation of the gene body is associated with a higher level of gene expression in proliferative cells [23, 26]. Indeed, these regions were overrepresented in our DMRs associated with gene elements (introns 26.7%; exons 13.6%). Moreover, it is worth mentioning that we found that 7.7%, 7% and 3.1% of these DMRs, were detected in promoters, first exons, and enhancers, respectively. DNA methylation located at promoter, the first exon and enhancer regions are often related to repression of transcription [24, 27, 28]. It is possible that transcription factors bind to DNA in a hypomethylated state, whereas they may not bind in the cells with methylated DNA [25, 28]. In addition, the GO enrichment analysis showed embryonic morphogenesis (GO:0048598) as enriched Gene ontology term, as well as some differences between gene elements. Specifically, the DMRs within 1 to 5 kb seem to be more related to genes with a role embryonic morphogenesis and neuron regulation, promoters to apoptotic processes, exons to cell organization, and introns to synapsis and dendritic associated processes. Regarding our results on DNA methylation in HSCR related genes already known, the most frequent differentially methylated gene element is gene body (exons and introns), although promoters, 5´UTR, 3´UTR and upstream regions (1 to 5 kb) were differentially methylated as well. Therefore, it seems that the methylated cytosines in different gene elements have different functions and future efforts aimed at deciphering the potential functional consequences of DNA methylation in DMRs related genes in HSCR patients are necessary.

In recent years, a growing number of studies have shown that non-coding RNAs (ncRNAs) play a significant role in HSCR onset [29]. Since the main goal of this study is to further clarify the epigenetic basis of HSCR, we decided to further examine the DMRs associated with ncRNAs. In this sense, we found that some DMRs genes are miRNA and lncRNAs, indicating that DNA methylation may affect ENS development by regulating other epigenetic factors. Among them, two lncRNA (*MEG3* and *AFAP1-AS*) and one miRNA (*MIR195*), have previously been identified as potential susceptibility genes for HSCR [29–32]. Moreover, it should be noted that these ncRNAs (*MEG3*, *AFAP1-AS* and *MIR195*) were included in the gene list of promising candidates according to the enrichment analysis. It should be mentioned that two differentially methylated genes encoded for lncRNAs previously described as potential genes with a role during ENS based on their expression in human EPCs (*IPW* and *NR2F1-AS1*) [29]. It has been reported an aberrant expression level of *MEG3*, *AFAP1-AS* and *MIR195* in gut tissues from HSCR patients, which suppressed cell migration and proliferation [30–32]. In addition, *MEG3* was also reported as potential susceptibility factor for HSCR by our group based on an increase at transcript level of *MEG3* in HSCR EPCs and potential pathogenic rare variants in its sequence carried by HSCR patients [29]. In the current study, an aberrant hypomethylation level was detected in *MEG3* and *AFAP1-AS*, *IPW* and *NR2F1-AS1*, whereas *MIR195* was detected as hypermethylated gene in HSCR EPCs. Therefore, the relationship of DNA methylation and ncRNA may be worth a more detailed study.

## Conclusions

We have carried out the first whole genome DNA methylation study of HSCR, which demonstrates that the genome of HSCR EPCs is predominantly hypomethylated. This study identified novel HSCR-related changes in DNA methylation that support an important role for epigenetics in ENS development and could also explain the incomplete penetrance observed in most HSCR cases. As the first step to elucidate the biological role of DNA methylation in EPCs, this data supplies important information for further insight into the regulatory functions of DNA methylation in HSCR.

## Methods

### Generation of NLBs from human EPCs

Postnatal human EPCs were extracted from ganglionic gut tissue of five non-related patients diagnosed with sporadic S-HSCR through the histopathological evaluation of a colon biopsy (male: female = 3:2). No variants in the HSCR associated genes have been detected in four of these patients. A RET variant (c.3185A > G, p.Tyr1062Cys, dbSNP: rs587778659) was detected in one of these patients, which was inherited from his healthy father [33]. Therefore, the presence of the variant in other unaffected relatives confirms its incomplete penetrance, and the study of other factors involved in the pathogenesis of the disease in this patient is still necessary. In addition, three patients with other gastrointestinal disorders such as anorectal malformations and enterocolitis were considered as controls (male: female = 1:2). The age range of the subjects studied was from 3 months to 3 years. The extraction and growth in culture of human EPCs were performed as previously described our group [6]. Written informed consent for surgery, clinical and molecular genetic studies was obtained from the guardians of all patients. The study was approved by the Ethics Committee for clinical research of the University Hospital Virgen del Rocío (Seville, Spain) and complies with the tenets of the declaration of Helsinki.

### Whole-genome bisulfite sequencing assay (WGBS)

#### Library preparation

DNA extraction from EPCs was performed by MasterPure™ Complete DNA and RNA Purification Kit (Lucigen, Wisconsin,USA). Then, Bisulfite conversion of genomic DNA samples (100 ng) was carried out using EZ DNA Methylation-LightningTM Kit (Zymo Research, California, USA). The quality of denatured DNA was evaluated using a RNA Pico Chip on Agilent 2100 Bioanalyzer (Agilent Technologies, USA). Library preparation of the extracted and bisulfite modified DNA was performed using TruSeq DNA Methylation kit (Illumina, USA). Libraries were assessed for quality assurance using the Qubit High-Sensitivity DNA kit on Agilent 2100 Bioanalyzer (Agilent Technologies, USA). Libraries typically showed a broad size distribution ~ 150–1000 bp, with median library sizes of 260–380 bp. All procedures were performed following the manufacturer's instructions.

#### Next-generation sequencing

Libraries generated with DNA fragments from five HSCR patients and three controls were pooled, diluted to a final concentration of 2.5 nM, and sequenced on the Illumina HiSeq3000 sequencer in 150 bp paired-end mode (Illumina, USA).

#### WGBS analysis

Reads were converted to fastq format using bcl2fastqc v. 2.19.1.403. Read quality was assessed through FastQC v0.11.7. Fastq Reads were trimmed with Trimmomatic v0.36 [34] with a minimum length cut off of 45 bp, and with a minimum quality cut off of 25 bp. After trimming, mapping and methylation calls were done using Bismark v0.19.1 [35] (GRCh38 as reference genome). DMRs were subsequently identified by dmrseq v0.99.0 [36] with a *p* *value* threshold of 0.05 in two sets of comparisons (HSCR and control cases). DMRs are regions (clusters of nearby CpGs) with systematic differences in methylation between groups, as compared to within-group variability. The package dmrseq provide an accurate two-stage method that detects candidate regions based on consistent evidence of differential methylation between groups as a first stage and evaluates statistical significance at the region level while accounting for known sources of variability as a second stage. Once candidate DMRs are identified, a statistical analysis for each candidate region is performed to estimate region-level statistics, which consider variability between biological replicates and spatial correlation among neighboring loci. Therefore, to identify candidate DMRs the methylation proportion difference (β) between samples is estimated from the proportion of methylated reads for each locus and condition. Local-likelihood smooth [37], which gives more weight to highly covered regions highly represented within a condition, is used to minimize false positive assignations. Thus, negative values correspond to hypomethylated regions, and positive values to hypermethylated regions. Significance of each region (p-value) was assessed by permutation testing of a pooled null distribution, which can be generated from as few as two biological replicates. FDR (q-value) is controlled using the procedure of Benjamini and Hochberg [38].

DMRs were annotated by annotatr v3.10 [39] to gene context and CpGs (CpG islands, CpG shores, CpG shelves, Cpg inter). Therefore, we defined 13 types of DMRs (cpg_islands/cpg_inter/cpg_shelves/cpg_shores/enhancers/1to5kb/3UTRs/5UTRs/exons/firstexons/introns/promoters/intergenic), which can be hypomethylated (ß < 0) or hypermethylated (ß > 0). According to the distance to CpG islands, different regions were considered, cpg_inter (more than 4 kb), cpg_shelves (2–4 kb) and cpg_shore (up to 2 kb). Each DMR was annotated based on its position in relation to all contexts previously mentioned; hence, one DMR can have multiple annotations. Additionally, in order to comprehensively analyze and interpret the data, the statistically enriched biological processes of each genomic annotation, as well as of all differentially methylated genes were calculated using Metascape [40]. Through this analysis, we first identified all statistically enriched GO terms, which were then hierarchically clustered into a tree based on Kappa-statistical similarities among their gene memberships. Then, 0.3 kappa score was applied as the threshold to cast the tree into term clusters. Then, a subset of representative terms from this cluster was selected and convert them into a network layout. The network is visualized with Cytoscape (v3.1.2). One term from each cluster is selected to have its term description shown as label.

#### Bibliography search

With the aim to gain a deeper understanding of how DNA methylation changes during ENS development may contribute to the onset of HSCR, we performed an automatic bibliographic search for the DMR genes detected in the WGBS analysis. To do this, a proprietary script that uses the Pubmed API to search for terms of interest in the abstracts available in Pubmed, was applied for each DMR gene (Hirschsprung disease and Enteric Nervous System) (Additional file 6: Text S1). Then, the selected papers were reviewed to select those genes with a solid role in any of the processes of interest based on expert judgment.

## Supplementary Information

**Additional file 1** **Table S1** Alignment with GRCh38 as the reference genome of all individuals (five HSCR patients and three controls).

**Additional file 2** **Table S2** DMRs identified in HSCR patients versus controls.

**Additional file 3** **Table S3** Top 20 enriched biological processes of the DMRs located according to different gene regions (1to5kb, promoters, exons, and introns). % is the percentage of all the user-provided genes that are found in the given ontology term. Log10(P) is the p-value in log base 10. Log10(q) is the multi-test adjusted p-value in log base 10.

**Additional file 4** **Table S4** Differentially methylated genes in HSCR patients versus controls.

**Additional file 5** **Table S5** Genes considered as promising candidates according to their biological processes. Genes in green were detected by GO analysis into the main enriched ontology clusters and previously described their involvement in HSCR. Genes in gray were detected by GO analysis into the main enriched ontology clusters and previously described their involvement in ENS.

**Additional file 6** **Text S1** Proprietary script that was used to search for terms of interest in the abstracts available in Pubmed by Pubmed API.

## Data Availability

The datasets analysed during the current study are available in the Sequence Read Archive (SRA) repository, http://www.ncbi.nlm.nih.gov/bioproject/683997, BioProject ID: PRJNA683997.

## References

[1] Chakravarti A, Lyonnet, S. In The Metabolic and Molecular Bases of Inherited Disease. In: Beaudet AR, Scriver, C. R., Sly, W., Valle, D., editor. 8th ed: McGraw-Hill; 2001.

[2] Luzón-Toro B, Villalba-Benito L, Torroglosa A, Fernández RM, Antiñolo G, Borrego S (2020). What is new about the genetic background of Hirschsprung disease?. Clin Genet.

[3] Amiel J, Sproat-Emison E, Garcia-Barcelo M, Lantieri F, Burzynski G, Borrego S (2008). Hirschsprung disease, associated syndromes and genetics: a review. J Med Genet.

[4] Lindley RM, Hawcutt DB, Connell MG, Edgar DH, Kenny SE (2009). Properties of secondary and tertiary human enteric nervous system neurospheres. J Pediatr Surg..

[5] Rauch U, Hänsgen A, Hagl C, Holland-Cunz S, Schäfer KH (2006). Isolation and cultivation of neuronal precursor cells from the developing human enteric nervous system as a tool for cell therapy in dysganglionosis. Int J Colorectal Dis.

[6] Ruiz-Ferrer M, Torroglosa A, Nunez-Torres R, de Agustin JC, Antinolo G, Borrego S (2011). Expression of PROKR1 and PROKR2 in human enteric neural precursor cells and identification of sequence variants suggest a role in HSCR. PLoS ONE.

[7] Torroglosa A, Villalba-Benito L, Fernandez RM, Moya-Jimenez MJ, Antinolo G, Borrego S (2017). Dnmt3b knock-down in enteric precursors reveals a possible mechanism by which this de novo methyltransferase is involved in the enteric nervous system development and the onset of Hirschsprung disease. Oncotarget.

[8] Lake JI, Heuckeroth RO (2013). Enteric nervous system development: migration, differentiation, and disease. Am J Physiol Gastrointest Liver Physiol.

[9] Torroglosa A, Villalba-Benito L, Luzon-Toro B, Fernandez RM, Antinolo G, Borrego S (2019). Epigenetic mechanisms in hirschsprung disease. Int J Mol Sci..

[10] Torroglosa A, Alves MM, Fernández RM, Antiñolo G, Hofstra RM, Borrego S (2016). Epigenetics in ENS development and Hirschsprung disease. Dev Biol.

[11] Okano M, Bell DW, Haber DA, Li E (1999). DNA methyltransferases Dnmt3a and Dnmt3b are essential for de novo methylation and mammalian development. Cell.

[12] Jin B, Tao Q, Peng J, Soo HM, Wu W, Ying J (2008). DNA methyltransferase 3B (DNMT3B) mutations in ICF syndrome lead to altered epigenetic modifications and aberrant expression of genes regulating development, neurogenesis and immune function. Hum Mol Genet.

[13] Martins-Taylor K, Schroeder DI, LaSalle JM, Lalande M, Xu RH (2012). Role of DNMT3B in the regulation of early neural and neural crest specifiers. Epigenetics.

[14] Torroglosa A, Enguix-Riego MV, Fernandez RM, Roman-Rodriguez FJ, Moya-Jimenez MJ, de Agustin JC (2014). Involvement of DNMT3B in the pathogenesis of Hirschsprung disease and its possible role as a regulator of neurogenesis in the human enteric nervous system. Genet Med.

[15] Villalba-Benito L, Torroglosa A, Fernández RM, Ruíz-Ferrer M, Moya-Jiménez MJ, Antiñolo G (2017). Overexpression of DNMT3b target genes during Enteric Nervous System development contribute to the onset of Hirschsprung disease. Sci Rep.

[16] Munnes M, Patrone G, Schmitz B, Romeo G, Doerfler W (1998). A 5'-CG-3'-rich region in the promoter of the transcriptionally frequently silenced RET protooncogene lacks methylated cytidine residues. Oncogene.

[17] Wang G, Zhang L, Wang H, Cui M, Liu W, Liu Y (2018). Demethylation of GFRA4 promotes cell proliferation and invasion in Hirschsprung disease. DNA Cell Biol.

[18] Tang W, Li B, Tang J, Liu K, Qin J, Wu W (2013). Methylation analysis of EDNRB in human colon tissues of Hirschsprung's disease. Pediatr Surg Int.

[19] de Pontual L, Trochet D, Bourdeaut F, Thomas S, Etchevers H, Chompret A (2007). Methylation-associated PHOX2B gene silencing is a rare event in human neuroblastoma. Eur J Cancer..

[20] Eckhardt F, Lewin J, Cortese R, Rakyan VK, Attwood J, Burger M (2006). DNA methylation profiling of human chromosomes 6, 20 and 22. Nat Genet.

[21] Lister R, Pelizzola M, Dowen RH, Hawkins RD, Hon G, Tonti-Filippini J (2009). Human DNA methylomes at base resolution show widespread epigenomic differences. Nature.

[22] Scelfo A, Fachinetti D (2019). Keeping the centromere under control: a promising role for DNA methylation. Cells..

[23] Arechederra M, Daian F, Yim A, Bazai SK, Richelme S, Dono R (2018). Hypermethylation of gene body CpG islands predicts high dosage of functional oncogenes in liver cancer. Nat Commun.

[24] Jones PA (2012). Functions of DNA methylation: islands, start sites, gene bodies and beyond. Nat Rev Genet.

[25] Volkov P, Bacos K, Ofori JK, Esguerra JL, Eliasson L, Rönn T (2017). Whole-genome bisulfite sequencing of human pancreatic islets reveals novel differentially methylated regions in type 2 diabetes pathogenesis. Diabetes.

[26] Aran D, Toperoff G, Rosenberg M, Hellman A (2011). Replication timing-related and gene body-specific methylation of active human genes. Hum Mol Genet.

[27] Brenet F, Moh M, Funk P, Feierstein E, Viale AJ, Socci ND (2011). DNA methylation of the first exon is tightly linked to transcriptional silencing. PLoS ONE.

[28] Ozanne SE, Constância M (2007). Mechanisms of disease: the developmental origins of disease and the role of the epigenotype. Nat Clin Pract Endocrinol Metab.

[29] Torroglosa A, Villalba-Benito L, Fernández RM, Luzón-Toro B, Moya-Jiménez MJ, Antiñolo G (2020). Identification of new potential LncRNA biomarkers in Hirschsprung disease. Int J Mol Sci..

[30] Li H, Li B, Zhu D, Xie H, Du C, Xia Y (2017). Downregulation of lncRNA MEG3 and miR-770-5p inhibit cell migration and proliferation in Hirschsprung's disease. Oncotarget.

[31] Chen G, Peng L, Zhu Z, Du C, Shen Z, Zang R (2017). LncRNA AFAP1-AS functions as a competing endogenous RNA to regulate RAP1B expression by sponging miR-181a in the HSCR. Int J Med Sci.

[32] Lei H, Tang J, Li H, Zhang H, Lu C, Chen H (2014). MiR-195 affects cell migration and cell proliferation by down-regulating DIEXF in Hirschsprung's disease. BMC Gastroenterol.

[33] Ruiz-Ferrer M, Fernandez RM, Antinolo G, Lopez-Alonso M, Eng C, Borrego S (2006). A complex additive model of inheritance for Hirschsprung disease is supported by both RET mutations and predisposing RET haplotypes. Genet Med.

[34] Bolger AM, Lohse M, Usadel B (2014). Trimmomatic: a flexible trimmer for Illumina sequence data. Bioinformatics.

[35] Krueger F, Andrews SR (2011). Bismark: a flexible aligner and methylation caller for Bisulfite-Seq applications. Bioinformatics.

[36] Korthauer K, Chakraborty S, Benjamini Y, Irizarry RA (2019). Detection and accurate false discovery rate control of differentially methylated regions from whole genome bisulfite sequencing. Biostatistics.

[37] Loader C (2006). Local regression and likelihood.

[38] Benjamini Y, Hochberg Y (1995). Controlling the false discovery rate: a practical and powerful approach to multiple testing. J Roy Stat Soc Ser B (Methodol).

[39] Cavalcante RG, Sartor MA (2017). annotatr: genomic regions in context. Bioinformatics.

[40] Zhou Y, Zhou B, Pache L, Chang M, Khodabakhshi AH, Tanaseichuk O (2019). Metascape provides a biologist-oriented resource for the analysis of systems-level datasets. Nat Commun.

