# Supplementary material for: Genome-wide analysis of DNA methylation in Hirschsprung enteric precursor cells: unraveling the epigenetic landscape of enteric nervous system development
Source: Clin Epigenetics. 2021 Mar 9;13:51. doi: 10.1186/s13148-021-01040-6 (PMC7942176; doi:10.1186/s13148-021-01040-6)
Supplement: Supplementary file 1 — Additional file 1 Table S1 Alignment with GRCh38 as the reference genome of all individuals (five HSCR patients and three controls). [file 13148_2021_1040_MOESM1_ESM.pdf]

Supplementary file 1: Table 1. Alignment with GRCh38 as the reference genome of all individuals (five HSCR patients and three controls)

	Total Reads	Aligned Reads	Percent Aligned Reads	Duplicate Reads (removed)	Unique Reads (remaining)	Fragment Length Median	CG Count	Fold Coverage
Control 1	33041173	26745640	80,95%	3184313	23561327	175 bp	69171607	2.353
Control 2	49499571	40004682	80,82%	6429764	33574918	156 bp	105109137	3.575
Control 3	36863030	29127931	79,02%	4344725	24783206	141 bp	74872261	2.546
HSCR 1	33735240	27193318	80,61%	4085059	23108259	152 bp	71806982	2.442
HSCR 2	29524704	24131505	81,73%	3311133	20820372	168 bp	63689740	2.166
HSCR 3	40480242	33186006	81,98%	6838678	26347328	168 bp	94232676	3.205
HSCR 4	35742472	28463581	79,64%	4445253	24018328	157 bp	79309911	2.697
HSCR 5	35213009	27842233	79,07%	4341852	23500381	160 bp	78039322	2.654
