# Supplementary material for: Genome-wide analysis of DNA methylation in Hirschsprung enteric precursor cells: unraveling the epigenetic landscape of enteric nervous system development
Source: Clin Epigenetics. 2021 Mar 9;13:51. doi: 10.1186/s13148-021-01040-6 (PMC7942176; doi:10.1186/s13148-021-01040-6)
Supplement: Supplementary file 3 — Additional file 3 Table S3 Top 20 enriched biological processes of the DMRs located according to different gene regions (1to5kb, promoters, exons, and introns). % is the percentage of all the user-provided genes that are found in the given ontology term. Log10(P) is the p-value in log base 10. Log10(q) is the multi-test adjusted p-value in log base 10. [file 13148_2021_1040_MOESM3_ESM.pdf]

Supplementary file 3: Table 3. Top 20 enriched biological processes of the DMRs located according to different gene regions (1to5kb, promoters, exons, and introns). % is the percentage of all the user-provided genes that are found in the given ontology term. Log10(P) is the p-value in log base 10. Log10(q) is the multi-test adjusted p-value in log base 10.

1to5kb	GO	Category	Description	%	Log10(P)	Log10(q)
1	GO:0048598	GO Biological Processes	embryonic morphogenesis	7.28	-10.36	-6.16
2	GO:0045664	GO Biological Processes	regulation of neuron differentiation	6.9	-7.88	-3.99
3	GO:0048729	GO Biological Processes	tissue morphogenesis	6.53	-7.01	-3.29
4	GO:0007423	GO Biological Processes	sensory organ development	5.6	-6.01	-2.7
5	GO:0030155	GO Biological Processes	regulation of cell adhesion	6.53	-6	-2.7
6	GO:0007420	GO Biological Processes	brain development	6.53	-5.7	-2.64
7	GO:0048705	GO Biological Processes	skeletal system morphogenesis	3.17	-5.66	-2.64
8	GO:0007169	GO Biological Processes	transmembrane receptor protein tyrosine kinase signaling pathway	6.34	-5.38	-2.46
9	GO:0035270	GO Biological Processes	endocrine system development	2.24	-5.27	-2.38
10	GO:0043068	GO Biological Processes	positive regulation of programmed cell death	5.97	-5.25	-2.38
11	GO:0051493	GO Biological Processes	regulation of cytoskeleton organization	5.04	-4.95	-2.25
12	GO:0060485	GO Biological Processes	mesenchyme development	3.36	-4.85	-2.19

13	GO:0045665	GO Biological Processes	negative regulation of neuron differentiation	2.8	-4.49	-1.95
14	GO:0050808	GO Biological Processes	synapse organization	4.1	-4.42	-1.92
15	GO:2000027	GO Biological Processes	regulation of animal organ morphogenesis	2.99	-4.41	-1.92
16	GO:0001101	GO Biological Processes	response to acid chemical	3.54	-4.33	-1.87
17	GO:0007264	GO Biological Processes	small GTPase mediated signal transduction	4.48	-4.24	-1.81
18	GO:0043523	GO Biological Processes	regulation of neuron apoptotic process	2.61	-4.14	-1.73
19	GO:0007224	GO Biological Processes	smoothened signaling pathway	2.05	-4.02	-1.65
20	GO:1901699	GO Biological Processes	cellular response to nitrogen compound	5.22	-4	-1.64
**Promoters**	**GO**	**Category**	**Description**	**%**	**Log10(P)**	**Log10(q)**
1	GO:0018958	GO Biological Processes	phenol-containing compound metabolic process	2.69	-5.69	-1.5
2	GO:0043524	GO Biological Processes	negative regulation of neuron apoptotic process	2.96	-5.28	-1.43
3	GO:0048598	GO Biological Processes	embryonic morphogenesis	6.18	-5.16	-1.43
4	GO:0030036	GO Biological Processes	actin cytoskeleton organization	6.72	-4.97	-1.37
5	GO:0043065	GO Biological Processes	positive regulation of apoptotic process	6.45	-4.71	-1.29
6	GO:1901615	GO Biological Processes	organic hydroxy compound metabolic process	5.65	-4.68	-1.29

7	GO:0002274	GO Biological Processes	myeloid leukocyte activation	6.18	-4.48	-1.29
8	GO:0007423	GO Biological Processes	sensory organ development	5.65	-4.44	-1.29
9	GO:0000904	GO Biological Processes	cell morphogenesis involved in differentiation	6.45	-4.09	-1.09
10	GO:2000147	GO Biological Processes	positive regulation of cell motility	5.38	-3.82	-0.96
11	GO:0017000	GO Biological Processes	antibiotic biosynthetic process	1.08	-3.81	-0.96
12	GO:1903827	GO Biological Processes	regulation of cellular protein localization	5.11	-3.81	-0.96
13	GO:0030155	GO Biological Processes	regulation of cell adhesion	6.18	-3.8	-0.96
14	GO:0048705	GO Biological Processes	skeletal system morphogenesis	2.96	-3.59	-0.85
15	GO:0045647	GO Biological Processes	negative regulation of erythrocyte differentiation	0.81	-3.58	-0.85
16	GO:0072273	GO Biological Processes	metanephric nephron morphogenesis	1.08	-3.44	-0.84
17	GO:0051345	GO Biological Processes	positive regulation of hydrolase activity	6.18	-3.44	-0.84
18	GO:0034330	GO Biological Processes	cell junction organization	3.49	-3.43	-0.84
19	GO:0043434	GO Biological Processes	response to peptide hormone	4.3	-3.42	-0.84
20	GO:0002702	GO Biological Processes	positive regulation of production of molecular mediator of immune response	1.88	-3.39	-0.84
**Exons**	**GO**	**Category**	**Description**	**%**	**Log10(P)**	**Log10(q)**

1	GO:0098742	GO Biological Processes	cell-cell adhesion via plasma-membrane adhesion molecules	3.62	-7.75	-3.72
2	GO:0007169	GO Biological Processes	transmembrane receptor protein tyrosine kinase signaling pathway	6.55	-7.62	-3.72
3	GO:0007423	GO Biological Processes	sensory organ development	5.43	-7.29	-3.69
4	GO:0007264	GO Biological Processes	small GTPase mediated signal transduction	5.01	-7.26	-3.69
5	GO:0000904	GO Biological Processes	cell morphogenesis involved in differentiation	6.41	-7.19	-3.69
6	GO:0018108	GO Biological Processes	peptidyl-tyrosine phosphorylation	4.04	-6.79	-3.51
7	GO:0098662	GO Biological Processes	inorganic cation transmembrane transport	6.27	-6.55	-3.46
8	GO:0030155	GO Biological Processes	regulation of cell adhesion	5.99	-6.21	-3.26
9	GO:0043087	GO Biological Processes	regulation of GTPase activity	4.46	-5.96	-3.04
10	GO:0007416	GO Biological Processes	synapse assembly	2.51	-5.86	-2.99
11	GO:0051129	GO Biological Processes	negative regulation of cellular component organization	5.99	-5.65	-2.8
12	GO:0048598	GO Biological Processes	embryonic morphogenesis	5.01	-5.54	-2.71
13	GO:0043062	GO Biological Processes	extracellular structure organization	4.18	-5.49	-2.7
14	GO:0030036	GO Biological Processes	actin cytoskeleton organization	5.57	-5.49	-2.7
15	GO:0007626	GO Biological Processes	locomotory behavior	2.51	-5.38	-2.66

16	GO:0048729	GO Biological Processes	tissue morphogenesis	5.29	-5.31	-2.62
17	GO:0043009	GO Biological Processes	chordate embryonic development	5.15	-5.24	-2.57
18	GO:0050732	GO Biological Processes	negative regulation of peptidyl-tyrosine phosphorylation	1.25	-4.93	-2.29
19	GO:0010942	GO Biological Processes	positive regulation of cell death	5.57	-4.91	-2.28
20	GO:1901137	GO Biological Processes	carbohydrate derivative biosynthetic process	5.71	-4.88	-2.27
**Introns**	**GO**	**Category**	**Description**	**%**	**Log10(P)**	**Log10(q)**
1	GO:0007156	GO Biological Processes	homophilic cell adhesion via plasma membrane adhesion molecules	4.49	-30.15	-25.95
2	GO:0120039	GO Biological Processes	plasma membrane bounded cell projection morphogenesis	7.25	-18.46	-14.74
3	GO:0050808	GO Biological Processes	synapse organization	5.18	-15.64	-12.34
4	GO:0050804	GO Biological Processes	modulation of chemical synaptic transmission	4.92	-13.01	-9.81
5	GO:0007423	GO Biological Processes	sensory organ development	5.26	-10.59	-7.74
6	GO:0007420	GO Biological Processes	brain development	6.13	-9.76	-6.94
7	GO:0007264	GO Biological Processes	small GTPase mediated signal transduction	4.66	-9.5	-6.71
8	GO:0016358	GO Biological Processes	dendrite development	2.93	-9.06	-6.3
9	GO:0007610	GO Biological Processes	behavior	5	-8.41	-5.66

10	GO:0044057	GO Biological Processes	regulation of system process	5.09	-8.37	-5.65
11	GO:0043087	GO Biological Processes	regulation of GTPase activity	4.23	-8.12	-5.42
12	GO:0098662	GO Biological Processes	inorganic cation transmembrane transport	5.78	-8.08	-5.39
13	GO:0007169	GO Biological Processes	transmembrane receptor protein tyrosine kinase signaling pathway	5.61	-7.66	-5
14	GO:0030036	GO Biological Processes	actin cytoskeleton organization	5.35	-7.56	-4.91
15	GO:1905114	GO Biological Processes	cell surface receptor signaling pathway involved in cell-cell signaling	4.83	-7.18	-4.57
16	GO:0010817	GO Biological Processes	regulation of hormone levels	4.4	-7.1	-4.51
17	GO:0048598	GO Biological Processes	embryonic morphogenesis	4.57	-6.63	-4.1
18	GO:0034762	GO Biological Processes	regulation of transmembrane transport	4.4	-6.45	-3.94
19	GO:0032870	GO Biological Processes	cellular response to hormone stimulus	4.92	-6.38	-3.88
20	GO:0042733	GO Biological Processes	embryonic digit morphogenesis	1.12	-5.99	-3.54
