# Supplementary material for: Genome-wide analysis of DNA methylation in Hirschsprung enteric precursor cells: unraveling the epigenetic landscape of enteric nervous system development
Source: Clin Epigenetics. 2021 Mar 9;13:51. doi: 10.1186/s13148-021-01040-6 (PMC7942176; doi:10.1186/s13148-021-01040-6)
Supplement: Supplementary file 6 — Additional file 6 Text S1 Proprietary script that was used to search for terms of interest in the abstracts available in Pubmed by Pubmed API. [file 13148_2021_1040_MOESM6_ESM.pdf]

Supplementary file 6: Text 1. Proprietary script that was used to search for terms of interest in the abstracts available in Pubmed by Pubmed API.

```
#!/usr/bin/env python

# coding: utf-8


import sys

import urllib

import xmltodict

import os

import re

import pandas as pd

import requests

import json

import numpy as np


base = '/mnt/lustre/scratch/CBRA/collaborations/ATorroglosa/PUBMED_API/'

gl = pd.read_csv(base + 'gene_list.txt', sep='\t', encoding='utf-8', low_memory=False, header=None)

st = pd.read_csv(base + 'search_term.txt', sep='\t', encoding='utf-8', low_memory=False, header=None)


search = pd.DataFrame()

array = []

n=0

error = []

for g in gl[0]:

    n=n+1

    print('\r' + str(g) + '\t' + str(n), end='')

    for s in st[0]:

        url = "https://eutils.ncbi.nlm.nih.gov/entrez/eutils/esearch.fcgi?db=pubmed&retmax=10&retmode=json&term=" + str(g) + "+AND+" + "\"" + str(s) + "\""

        response = requests.get(url)
```

```
        data = response.json()
        if 'esearchresult' in data.keys() and 'idlist' in data['esearchresult'].keys():
            for i in data['esearchresult']['idlist']:
                array.append(pd.DataFrame.from_dict({'pubid' : [i], 'gene' : [g], 'search' : [s]}))
        else:
            error.append(g)
            print(str(g) + ' error: ' + str(data))


search = pd.concat(array)
search.set_index('pubid', inplace=True)


n=0
for i in search.index:
    n=n+1
    print('\r' + str(i) + ' -- ' + str(n), end='')


    if os.path.isfile(i + '.xml'):
        with open('./temp/' + i + '.xml', 'r', encoding='utf8') as xml_file:
            file = xml_file.read()
        xml = xmltodict.parse(file)
    else:
        url = "https://eutils.ncbi.nlm.nih.gov/entrez/eutils/efetch.fcgi?db=pubmed&id=" + str(i) +
"&retmode=xml&rettype=abstract"
        response = requests.get(url)
        with open('./temp/' + i + '.xml', 'w') as f:
            f.write(response.text)
        xml = xmltodict.parse(response.content.decode('UTF-8'))


    if list(xml['PubmedArticleSet'].keys())[0] == 'PubmedArticle':
        dd = xml['PubmedArticleSet']['PubmedArticle']['MedlineCitation']['Article']
    elif list(xml['PubmedArticleSet'].keys())[0] == 'PubmedBookArticle':
        dd = xml['PubmedArticleSet']['PubmedBookArticle']['BookDocument']
    else:
```

```
    print('Error parsing' + i)
    continue

 #############
# Parse Title
if isinstance(dd['ArticleTitle'], dict):
    search.loc[i, 'title'] = dd['ArticleTitle']['#text']
elif isinstance(dd['ArticleTitle'], str):
    search.loc[i, 'title'] = dd['ArticleTitle']
else:
    print('Error:' + i + ' no title')

################
# Parse Abstract
if 'Abstract' not in dd.keys():
    print('Error:' + i + ' no abstract')
    continue



if isinstance(dd['Abstract']['AbstractText'], dict):
    if '#text' in dd['Abstract']['AbstractText'].keys():
        search.loc[i, 'abstract'] = dd['Abstract']['AbstractText']['#text']
    elif 'b' in dd['Abstract']['AbstractText'].keys():
        search.loc[i, 'abstract'] = dd['Abstract']['AbstractText']['b']
    else:
        print('Error:' + i + ' no abstract')

elif isinstance(dd['Abstract']['AbstractText'], list):
    ab = ''
    for ind in dd['Abstract']['AbstractText']:
        if isinstance(ind, str):
            ab = ab + ind + ' '
```

```
                continue
            if '#text' not in ind.keys():
                print('Error:' + i + ' no abstract')
                continue
            if '@label' not in ind.keys():
                ab = ab + ' ' + ind['#text'] + ' '
                continue
            ab = ab + ind['@Label'] + ': ' + ind['#text'] + ' '
        search.loc[i, 'abstract'] = ab
    else:
        search.loc[i, 'abstract'] = dd['Abstract']['AbstractText']

len(search['gene'].unique())

search.to_csv('pubmed_search_results.tsv', sep='\t')

search['id'] = search.index

gb = search.groupby(['gene', 'search', 'id']).first()

gb.to_csv('pubmed_search_groups.tsv', sep='\t')

df = pd.read_csv('pubmed_search_results.tsv', sep='\t')

len(df['gene'].unique())
```
